# Disability and the Medical Posthumanities

**DOI:** 10.26522/posthumanismjournal.v2i2.4341

**Published:** 2023-11-07

**Authors:** Dan Goodley

**Affiliations:** iHuman, School of Education, University of Sheffield

## Abstract

This paper makes a case for *being in* but *not of* the medical posthumanities, cognisant of our contemporary times that continue to render some human beings as valued and others as expendable. I provide a brief reading of medical posthumanities before turning to a *field* (critical disability studies), an *event* (the deployment of Do Not Attempt Cardiopulmonary Resuscitation notices to disabled people during the Covid-19 pandemic in the UK) and a *respons*e (reflected in the activism of People First, the international movement of people with learning disabilities). I contemplate some tensions that emerge when the field, event and response rub up against the medical posthumanities, working with the humanist register, more-than-human possibilities, and human troubles. I conclude with the argument that unless the medical humanities engage with disability then they are in danger of ‘ability-washing’ their research and scholarship.^1^

## Introduction

In recent years we have witnessed a growing intellectual and research community associated with the medical posthumanities. This approach studies health and well-being not solely as a human concern, but one wrapped up in the co-dependencies of multiple non/human actors and forces (Cohn and Lynch, 2017; Andrews, 2019; Gibson et al, 2021). Human predicaments are intimately connected to their relationships with other humans and non-human animals, and with machines and environments, spanning material, immaterial, biological, cultural, social, digital, planetary, spiritual, living, organic, and dead aspects of life. By acknowledging death as well as life the posthumanities recognise the importance of both. By positing (on an ontological perspective) that non/human objects and agents reside on the same level of existence, posthumanism considers humans and non-humans to be of equal importance as they co-evolve in ways that manifest a lack of clear distinction between them (Andrew, 2019: 1113). Moreover, following Dolezal *et al*. (2021), a posthuman move has been driven in part by a disenchantment with healthcare systems and healthcare research that are tied to a neoliberal emphasis on human self-sufficiency, personal responsibility, and market-based systems. The ‘practical impossibility’, Dolezal et al (2021: 7) write, ‘inherent in such idealized individualism has contributed to a growing awareness of how human beings are inextricably interwoven and interdependent with others – human and non-human; organic and inorganic – and that all life is reliant on these entanglements’. The medical posthumanities have the potential to radically reconfigure hierarchical structures between and within species and create spaces for contemplating ever-changing embodiments and modes of belonging, where health is always caught up in the entanglements of humans, non-humans, and planetary life (Dolezal et al, 2021: 15). For Piennar et al (2021), health and well-being are best considered in terms of their more-than-human relationalities and this appreciation has been informed by the critical posthumanities (e.g. Braidotti, 2013, 2019a, 2019b, 2020; Braidotti and Hlavajova, 2018). One of the theoretical offerings of the critical posthumanities lies in its focus on the human subject’s place in what Serres (2003) terms a ‘logic of relations’. Humanness is always contextual, relational and distributed and our co-dependence with non/human others is a defining feature of what makes us human. This focus on non/human assemblages is also under-girded by a posthuman emphasis on affirmative ethics: a fresh approach to research and scholarship that seeks to drop academia’s tendency for nihilistic deconstructionism (Bargués-Pedreny, 2019). Instead, an affirmative perspective seeks out hope, possibility and productivity in the many non/human entanglements that characterise our contemporary lives (Braidotti, 2019b).

At times it feels like contemporary academic discourse has been seduced by the posthumanities. Many of us relate to the pull and push of our interconnectedness with animals, ecosystems, technology, machines, science, and other humans. Who doesn’t want to be posthuman, a cyborgian citizen, a complex merging of hardware and wetware, a chimera of human and robotic potential? While I have no desire for space travel (nor the funds) - and am still getting my head around my iPhone, Twitter and Alexa (it’s an age thing) - non/human entanglements appear to be easier to access for some than others. Here, I am not suggesting that the medical posthumanities share the same imaginary register as that of Elon Musk. What I am suggesting is that we should not uncritically accept the seductive and promissory discourses of the posthumanities. What is to be done when some of our fellow human beings - and certain humanities - continue to be actively diminished even in these affirmative times of the post-Anthropocene? If the cyborg is one positive figuration of the promise of the posthumanities then the Covid-19 pandemic is the ultimate death-making consequence of our posthuman times. Covid-19’s zoonotic status has demonstrated beyond any doubts the global and local ways in which health and disease are reproduced at the complex interface of ‘human-animal-ecosystems’: a state of affairs that has been described as ‘One Health’ (e.g. Schwabe, 1984; FAO, OIE, WHO, 2010). The pandemic can be read as a specific consequence of the convergence of the fourth industrial revolution, the sixth extinction and a particular moment in the Capitalocene, characterized by the insatiable greed of consumer society, interpenetrations of global capital and the exploitation of animals and environments (Braidotti, 2020). As Hayles (2021a: s72) argues, perhaps *now* we have the opportunity to reflect on the concepts, vocabularies, imaginations and practices that sufficiently capture the complex interdependencies of humans, animals and ecosystems, and, crucially, to ‘rethink the ways in which we can identify with each other and with life forms radically different from us’. We should pause here to recognise that the opportunity to reflect, rethink and recalibrate is a privilege accorded to those of us with the luxuries of time, money, vaccines and healthcare systems. For others, well, survival is the only name of the game.

By suggesting that human and non-human objects and agents reside on the same level of existence, certain iterations of posthuman medical humanities appear to contemplate non/humans as having equal importance as they co-evolve in ways that lack clear distinction (Andrews, 2019: 1113).

There are multiple other examples of what you would call prototypes of collective assemblages, moving in the direction of the actualisation of the virtual. There are some well-established assemblages, and my favourite, because it is my political culture, is the alliance of the marginals. These are the queer, post-human missing people that my heart goes out to: women, feminists, LGBTQ+, animals, illegal unregistered migrants, disabled people, but also the non-human agents, like dust, plastic, dying insects and the Earth-others (Braidotti, 2019b: 470).

This flattening of the distinctions between non/humans does, however, raise a number of ethical, theoretical, and political concerns. There are clear tensions in advocating for a shared accountability to humans, animals, and the planet when there are complex and often competing histories and economies of non/human exclusion and exploitation.

The Covid-19 pandemic demanded that we all exercise what Hayles (2021b) describes as humility, caution, and respect for non/humans as we contemplate human evolution. Similarly, Kim (2015: 20-21) argues that thinking across human and non-human life opens us up to ‘new ways of imagining ourselves in relation to others…to develop a critical and transformational politics, to radically restructuring our relationships with other humans, animals and the environment’. Concurrently, we do not know enough about the sheer amount and ecological impact of pandemic litter and waste such as masks, testing materials, gloves, hand sanitiser bottles and personal protective equipment. A common matter emblematic of pandemic waste is plastic – or synthetic organic polymers – which has become a major marker of the Anthropocene (Pathak and Nichter, 2019). In the summer of 2020, Greenpeace reported that disposable surgical face masks – many containing plastics that are deemed unrecyclable because of potential contamination – pose short-term and long term social and environmental risks, as floating marine debris impacts on marine ecosystems and increased incinerator usage pollutes (Yeh, 2020). These new forms of Covid-19 waste heighten what has already been described as a global crisis to ecosystem health (Law et al, 2020; Arnold, 2021). Focusing on Covid-19 commodities responds to scholarship across the arts, humanities and social sciences that increasingly understands more-than-human objects as effective vehicles for exploring relations between culture, society and economy. As Bridge and Smith (2003) argue, commodities are produced for exchange and bestowed with various social meanings. Hence, objects have lives, biographies and careers that are reproduced through circulating transnational non/human networks of reproduction and consumption. Thinking of these non-human objects in these relational ways permits us to explore the fabrication, use, meaning, disposal, and recycling of a host of pandemic materials, to render the geographical life of a commodity more transparent (Bridge and Smith, 2003). One can extend this posthuman sensibility to an analysis of the commodification of non-human animals: productive forms of nonhuman life that are now put to work alongside us (Johnson, 2017). Animals are becoming ever-more expendable in the production and testing of vaccines (Bailone et al, 2020), recategorised as threats or resources (Hobbs and Reid, 2020) and subject to unethical and inhumane breeding programmes to meet human health demands. The pandemic also brought with it a spike in ownership of companion animals, a relationship that is closely linked to notions of improving human mental health during periods of lockdown (McDonald and Otte, 2020). Animals are both disposable and expendable; following Mansfield (2003) there is a need to examine how animals are transformed into commodities as a response to Covid-19, transported across different national spaces and incorporated into new cultural practices.

The pandemic has also illuminated, in horrific ways, how various nation states respond to their poor, disabled, sick, elderly, homeless and racialised citizens, rendering these humans invisible, disposable or excommunicated from the sphere of human concern (Giroux, 2006).^2^ The failure of health systems to provide equitable care threatens to further exacerbate a human crisis, making some human beings less-than-human (Braidotti, 2020). These failures are illuminated by the sheer numbers of humans killed by Covid-19. As Giroux (2006: 174) writes, ‘cadavers have a way of insinuating themselves in consciousness, demanding answers to questions that aren’t often asked’. Social theorists and public intellectuals have attended to these matters of human life and death, unearthing the many reasons why some human beings are afforded positions of privilege while others are rendered wasted, precarious, bare, dead, disposable or debilitated (Giroux, 2006; Mbembe, 2003; Agamben, 1998; Bauman, 2004; Butler, 2004, Puar, 2009). One wonders how the anthropocentric positionality within much social theory across the humanities, social and human sciences sits with the more-than-human preoccupations of the medical posthumanities. Furthermore, I remain anxious about the move *beyond* the human – towards the more-than-human – when there is still clearly much work to be done in interrogating how the human category has been historically and hegemonically assumed, evoked, and deployed in deeply exclusionary ways.

Even in these heady times of our posthuman age, the health and well-being of certain sections of human society continue to suffer. Hence, for the rest of this paper, I will sit with some of these inequalities in order to appraise posthuman perspectives of their value, worth and application. For the remaining sections of this paper I turn to a *field* (critical disability studies), an *event* (the deployment of Do Not Attempt Cardiopulmonary Resuscitation notices to disabled people during the Covid-19 pandemic in the UK), and a *response* (reflected in the activism of People First; the international movement of people with learning disabilities). By centering disability, I consider what is gained and lost by the posthuman turn. I contemplate three tensions: working the humanist register, more-than-human possibilities, and human troubles. I conclude that there is an urgent need to address ‘ability-washing’ in medical posthumanities through a thorough engagement with disability and critical disability studies.

## A field

Critical disability studies is an interdisciplinary field of inquiry that foregrounds the lives and aspirations of disabled people and their relationships with wider society. The last 40 years have witnessed the emergence of an established interdisciplinary field (Meekosha and Shuttleworth 2009; Mallett and Runswick-Cole, 2014; Goodley, 2014, 2016).The theoretical maturity, methodological advancement and practical utility of this scholarship is testimony to disabled activists, researchers and practitioners positioning disability as *the* central concern of research, policy and practice. There are over 1 billion disabled people in the world (WHO/World Bank, 2011), constituting the world’s biggest minority group, and a global community that has expanded as a consequence of the pandemic. Disability is also a complex mix of biological, psychological, relational, social, historical, cultural, political, institutional, systemic, material, ecological and economic factors (Shakespeare, 2013). Distinct models of disability have emerged, in various national contexts, emphasising disability’s minority status (North America), socio-economic foundation (United Kingdom), cultural location (Australia and North America), relational constitution (Nordic countries), bio-psycho-social character (supranational perspectives such as World Health Organisation and United Nations) and postcolonial influences (including Africa, Asia and South America) (see Mallett and Runswick-Cole, 2014 for a useful overview of these different models). While critical disability studies has moved in many different intellectual and disciplinary directions, disability still endures a difficult relationship with the medical humanities. Critical disability researchers have long expressed concerns about the conventional ways in which disability and disabled people are conceptualised, represented and included within the empirical and theoretical work of social and humanities researchers (Thomas, 2007; Blume et al, 2014; Stoddard-Holmes, 2015). Critical disability studies often reside on the periphery of the medical humanities. Indeed, the disability critiques of medical sociology offered by Thomas (2021) and Maulden and Lewis (2021) can be readily applied to medical humanities. And when disability fails to be a central concern then there is a real risk of rendering disability invisible. When disability does enter the fray, however, then this is also not without problems. Herndl (2005) observes that because the medical humanities focus on medical ethics, knowledge and practice, this side-lines disability’s constitution in a number of other social, cultural and institutional locations. While recent iterations of medical humanities – including the medical posthumanities - have sought a broader engagement with the health humanities (Jones, 2017), disabled people still complain of being framed primarily as passive recipients of healthcare or objects of bioethical debate. The place of disability in medical humanities is captured well by Garden’s (2010) argument that,

Medical humanities scholars who incorporate disability studies and disability rights perspectives into their work can help clinicians and others to improve medical care for disabled people and those who are chronically ill. (page 74)

This idea of incorporation into clinical discourse and practice captures a place typically occupied by disability in medical humanities: an absent presence (Titchkosky, 2011). Disability is often present (as a medico-bureaucratic problem to be addressed) but also absent (as a research colleague or scholarly authority) (see also Blume et al, 2014). The under-representation of disabled scholars in the medical humanities reflects a wider systemic under-representation of disabled colleagues across the whole of the academy, and leading to an under- and misrepresentation of disability in the theory and empirical research (Brown and Leigh, 2020; Durban, 2022).

While the relationship between medical humanities and disability remains questionable, there has been a more thorough coming together of critical disability studies and the critical posthumanities (Mitchell and Snyder, 2015; Gibson, 2016; Taylor, 2017; Saur and Sidorkin, 2018; Lundblad, 2020; Narainan, 2020; Gibson et al, 2021; Braidotti, 2020). In many ways disability is *the* ideal subject for the medical posthumanities. Disability can be understood as *the* quintessential posthuman subjectivity because it calls for new ontologies, ways of relating and engagements across the non/human divide (Goodley et al, 2014). From service animals, to prosthetics, to networks of care, to adaptive and assistive technologies, disability’s history is a posthuman history. The posthuman turn within medical humanities should, in theory, provide ample opportunities for reconnecting with critical disability studies. This said, while these opportunities undoubtedly exist, they also create a number of tensions; some of which I will explore in this paper.

Before continuing my analysis, it is important to acknowledge my own positionality as a white, cisgendered, non-disabled male writing from the colonial centre of Brexit Britain. Non-disabled researchers such as I must always attend to their privilege and to check that they are working in collaboration with disabled people as co-researchers and co-authors of disability theory and inquiry (see Stone and Priestley, 1996). The vast majority of my own funded research projects adopt participatory and co-production models of research that centralise the contributions of disabled people as researchers, theorists and activists.^3^ While participatory models are crucial, this should not dissuade non-disabled researchers from interrogating their own conceptual and theoretical baggage. As Smith et al (2017) argue in their paper on white professors teaching racism, these same professors should never escape a critique of their own white privilege. Similarly, it is incumbent upon non-disabled researchers and scholars to not only acknowledge one’s ability privilege but also ensure that empirical research and theorisation attends to different processes of ableism and disablism. The *dis/ability complex* recognises the ways in which two distinct though complementary processes come together: *disablism* (the exclusion of people with physical, sensory and cognitive impairments) and *ableism* (the idealisation of able-bodied-and-mindness) converge (Thomas, 2017; Goodley, 2018; Goodley et al, 2021). I wonder to what extent medical posthumanities scholars have this complex in mind when they are contemplating our non/human relationalities.

A note on language. I adopt the term disabled people in this paper because it is the one preferred by many British activists with sensory, physical and cognitive impairments to recognise the fundamentally political nature of disability. To be disabled is to be excluded by wider society; identifying oneself as a disabled person recognises one’s experiences of disablism. In this, it is important to acknowledge my own localism. The term ‘people with disabilities’, for example, is adopted by many people in the States and other contexts in order to respect ‘people first’ language. Meanwhile, across many other social locations, people may well identify in impairment-specific ways (as Blind, Deaf, learning disabled, neuroatypical as examples). By referencing disabled people, we attend to disability’s central role in the constitution of modernity not least in the valuing and sorting of valued and preferred ontologies and forms of being human (Hughes, 1999). I also refer to ‘people with learning disabilities’ - the term preferred in Britain - aware that this phenomenon is understood differently in other contexts in terms of developmental, cognitive or intellectual disabilities. While adopting the term people with learning disabilities I will also consider how some people so-labelled prefer the term People First.

### An event

In February 2021 the British broadsheet *The Guardian* ran the following story under the headline ‘Fury at ‘do not resuscitate’ notices given to Covid patients with learning disabilities. Vulnerable people have encountered ‘shocking discrimination’ during pandemic, says Mencap charity’. It continued:

People with learning disabilities have been given do not resuscitate orders during the second wave of the pandemic, in spite of widespread condemnation of the practice last year and an urgent investigation by the care watchdog. Mencap said it had received reports in January from people with learning disabilities that they had been told they would not be resuscitated if they were taken ill with Covid-19. The Care Quality Commission said in December that inappropriate Do Not Attempt Cardiopulmonary Resuscitation (DNACPR) notices had caused potentially avoidable deaths last year. (Tapper, 2021: np).

Prior to the pandemic, people with learning disabilities already died 20-30 years earlier than their non-disabled counterparts (NHS, 2017, LeDeR, 2019). A number of high-profile scandals, inquiries and reports into Winterbourne View, Whorlton Hall, Mendip House, Slade House and Yew Trees Hospital have revealed the tragic consequences of dehumanising care for disabled people in general and adults with learning disabilities more specifically (see for example Ryan, 2017). By Autumn 2020, people with learning disabilities were six times more likely to die from Covid-19 than the rest of the population (Public Health England, 2020). Controversies associated with the Clinical Frailty Scale, healthcare rationing, the aforementioned ‘Do Not Resuscitate’ orders, the neglect of care homes and changes in guidance around the vaccination programme, had the cumulative effect of devaluing the human worth and value of people with learning disabilities.

These inequalities remind us that conceptualisations of health and well-being need to be understood in the context of the dis/ability complex. In general, disabled people – and, more specifically, people with learning disabilities – continue to be the recipients of health services that neglect or demonise them; an institutionalised and systemic bias magnified by the pandemic (see Ryan, 2019; Shakespeare et al, 2022). Disabled people risk being neglected by healthcare (disablism) or ignored by healthcare practices that are designed with only non-disabled people in mind (ableism). These dual processes of ableism and disablism were magnified by the pandemic. The implicit reliance of all nation states upon self-sufficiency, isolation and shielding assumed a particular kind of ableist standard citizen, one ready, willing and able to look after themselves and their families. Moreover, as the pandemic rampaged through various communities, this only exposed the already neglected and under-resourced services that had for many years failed numerous disabled patients and service users (Shakespeare et al, 2022).

This focus on the predicament of disabled people highlights my own anthropocentric preoccupations. This paper unapologetically seeks to centralise the human condition and asserts that our current societal epoch constitutes some people as less than human. I seek to write back into medical posthumanities through an engagement with scholarship that responds to these very real events of human exclusion: critical disability studies. In so doing, I have in mind an invitation by Paul Gilroy (2018: 21) to those engaged in or with posthuman theorising: ‘With these new initiatives in mind, I hope you will be prepared to join with the ongoing work of salvaging imperilled humanity from the mounting wreckage’. I share Adams and Weinstein’s (2020: 235) position of being ‘in but not of the posthuman turn’ and am interested in understanding the ways in which the ‘academic project of posthumanism can be seen as risking/depriving/sharing with the oppressed the humanity that those scholars presume’ (Ibid). When the health and well-being of disabled people continue to be matters of life and death then we have some serious critical work to do.

### A response

**Figure 1:**
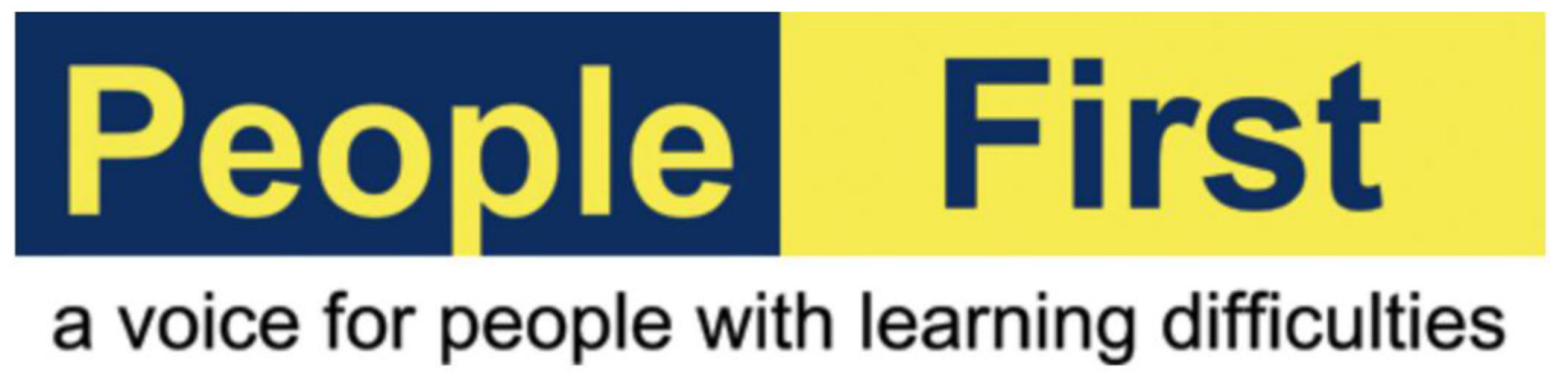
Logo for People First UK from https://peoplefirstltd.com

People with learning disabilities have, themselves, provided one response to their imperilled humanities: disability activism. Figure 1 is taken from the website of People First UK, an organisation run by and for people with learning disabilities.

**Figure 2:**
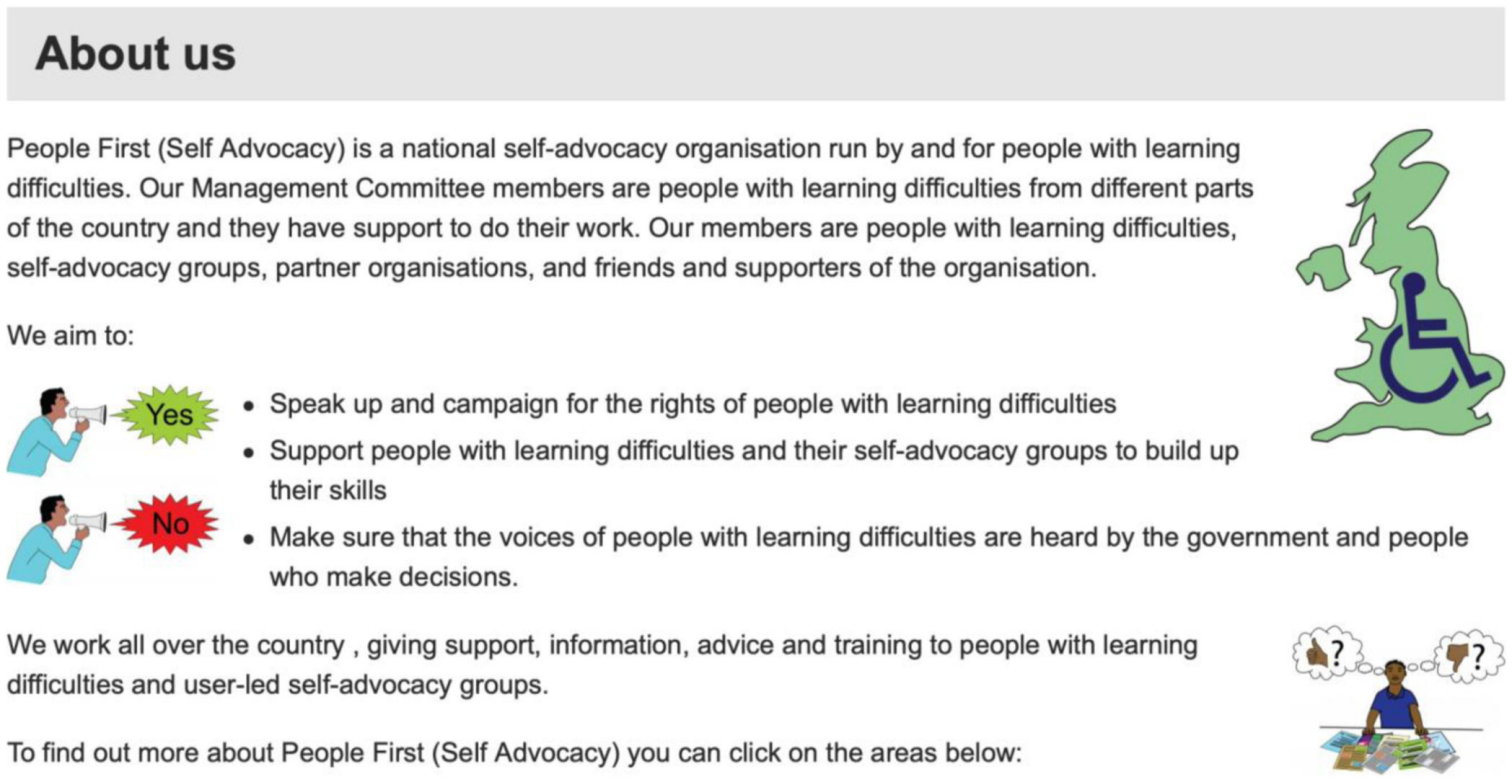
‘About us’ from People First website from https://peoplefirstltd.com

Figure 2 captures in text and images a brief description of this organisation and the wider international movement. The use of images and written text is deliberate, aimed at making the information accessible to people with learning disabilities who might not read.

When the pandemic hit the UK in 2020 many people with learning disabilities were already living precarious lives. People First and other self-advocacy groups responded immediately - including through the constitution of online communities and support networks. Many disabled people, and especially people with learning disabilities, experience digital poverty and online exclusion (Macdonald and Clayton, 2012). This material inequality had to be addressed. Self-advocacy groups mobilised their resources to equip people with learning disabilities in their local communities with the necessary hardware to access the online world, including smartphones and tablets. Armed with these new resources, many people were then able to access innovative modes of organisation as peer support and community engagement were created. Sunderland People First (2020), for example, set up daily meetings to address isolation and loneliness while Sheffield Voices (2020) hosted online Zoom coffee mornings to connect with people with learning disabilities across the city. In some cases, these connections were newly made and had not existed prior to the pandemic. Speakup Self-advocacy Rotherham (2020) moved quickly from supporting people to shield and stay-at-home to creating a new fully online mode of daily organising. Barod (2020), a workers cooperative, provided critical and constructive advice for allies and supporters of self-advocacy, to keep with a model of partnership even in the midst of the urgency provoked by the pandemic. For the next section of this paper, I sit with the politics of self-advocacy and some of the responses of self-advocacy groups to the event described above and consider how this activism rubs up against the medical posthumanities.

### Disability and Medical Posthumanities: three tensions

Sitting with the risk of erasure of their humanity (of the event) and the more affirmative aspects of their activism (the response) of people with learning disabilities, I now move into a critical reading of the medical posthumanities drawing upon ideas from critical disability studies (the field). I think it is possible to identify three tensions; i. working the humanist register; ii. More-than-human possibiltiies and iii. human troubles.

#### i Working the humanist register

One way out of the disablism and ableism inherent in contemporary constitutions of the human, and a key tenet of posthuman scholarship, is a rejection of humanism. Braidotti’s (2013) description of the humanist human is useful here as she notes, this ‘is very much a male of the species: it is a he’ (Ibid: 24). Moreover, ‘he is white, European, handsome and able-bodied’ (Ibid: 24), ‘an ideal of bodily perfection’ (Ibid: 13), ‘implicitly assumed to be masculine, white, urbanized, speaking a standard language, heterosexually inscribed in a reproductive unit and a full citizen of a recognised polity’ (Ibid: 65), ‘a rational animal endowed with language’ (Ibid: 141). Becoming posthuman might be viewed as a reaction to this exclusionary conception of humanity: to seek new ways of being human with other humans and non-humans. Nevertheless, there remains a humanist imperative to the language, tactics and strategies of People First, a sense of *working the humanist register*. This is hardly surprising when this collective is organising within economic, structural, cultural and jurisprudence systems that are underpinned by the hegemony of humanism. People First remind us that while posthumanism might be in vogue, humanism continues to dominate the language and politics of recognition. People First make use of categorisations, meanings and signifiers of the humanist human. People First appeals to ideas that are already known in the world – *people* – and makes a case for recognition in relation to these phenomena. People First exists as a counter-hegemony to the systemic dehumanisation of disabled people (the erasure of humanness). That fellow human beings have to utilise such stark assertions that their lives and personhood do matter is testimony to the stubborn grip of ableism and disablism. People First calls out for recognition of personhood that exists before and beyond the label of learning disabilities. If Tapper’s (2021) piece on DNACPR captures the problems then People First provides a solution and one very much humanist in nature.

To understand the significance of the work of People First we require a theoretical and philosophical interrogation of the constitution of the human condition. Here we can draw on critical disability studies’ engagements with Black Studies, specifically the notion of sociogeny developed by Fanon (1993) – and elaborated by Wynter (1992, 2003, 2006) – that refers to the study of the development of a social phenomenon. Goodley et al (2020: 138) write that ‘in counter-distinction to phylogeny (the study of evolution of the species) and ontogeny (the biological development of an individual organism) – a sociogeny unpacks the social, historical and cultural constitution of race and humanness (see Gagne, 2007 for a helpful overview)’. When we read disability through the methodologies of phylogeny or ontogeny, we reduce disability to accounts of evolution or biology. Sociogeny approaches the study of disability, and associated phenomena such as health and well-being, in terms of its fundamentally social, cultural, political and psycho-social constitution. Any conceptualisation of the activist language and practice undertaken by People First has, therefore, to be understood in sociogenic terms, i.e. as a matter of reclaiming the lives of those whose health, worth, value and well-being have been historically endangered and compromised by processes of racism, colonialism, medicalisation and disablism. These forms of exclusion work, in part, by constituting disabled people as antithetical to the humanist human. The preferred citizen of late capitalism is humanist man, the autonomous, fully evolved, eugenic or able, biocentric and *homo oeconomicu*s human, a human described in ‘the ethno-class terms of Darwinian Man’ *which is then* ‘*over-presented as the human*’ (Wynter, 2006: 128, italics added). This human category has been created by ‘the West’s institutionalization of itself in terms of its then epochally new self-conception or sociogenic code as Absolute Being’ (Wynter, 2006: 146). In contrast, disability endures being constituted as an ‘unbearable wrongness of being’ (Wynter, 2006:114) – the direct opposite of contemporary interests of Western, White, Bourgeois Man. Yet, in order to make their case, People First members are propelled to participate in a paradoxical engagement with humanism, pulling on the language of humanism (lives and people) while at the same time drawing attention to humanism’s inbuilt exclusionist tendencies to render some people as less than human (disabled). Here is the rub of a frictional relationship with humanist hegemony. As Adam and Wienstein (2020: 237-238) put it, ‘how do we come to know ourselves outside of the ontologies that have dictated who we are or the dominant knowledge system that has operationalized what it means to be human?’ Thinking with People First necessarily demands a sociogenic response, unpacking the disablism and ableism ‘embedded in the discussions that seek to transcend the human’ (Adam and Weinstein, 2020: 236, my italics) while simultaneously attending to the *ability expectations* implicit in many posthuman theorisations that mirror the ‘human ideal according to Western civilization (as a human, agent, subject, citizen, consumer, or worker)’ (Kögel and Wolbring, 2020: 237, my italics). Any commitment to medical posthumanities must be one that firmly rejects whiteness and able-bodied-and-mindedness as assumed elements of preferred citizenry and personhood. But our commitment should also remain mindful of the ways in which humanism informs the socio-cultural symbolic and, by extension, infiltrates the discourse of disability activism. We are reminded, here, of Mitchell and Snyder’s (2015) concept of the ‘able-disabled’ where disability (as with blackness) is invited into the centre of cultural logics when disabled people appropriate the language of humanism: hard working, responsible, self-sufficient, autonomous, independent. This is not to accuse People First of kowtowing to a respectable, toothless, reasonable version of politics and recognition; more an appeal to recognise that humanist discourse frames how the human is understood and valued and that activists are forced to work within this echo-chamber^4^.

#### ii More-than-human possibilities

We now turn attention to a second tension in posthuman health studies, one we might term *more-than-human possibilities*. The continued denigration of disabled human life, amplified by the Covid-19 pandemic, takes place in the context of ‘One Health’, the reproduction of health and disease at the human-animal-ecosystem interface (e.g. Schwabe, 1984; FAO, OIE, WHO, 2010). As mentioned earlier, critical disability studies have been quick to critically engage with this interface, pitching disability as *the* quintessential posthumanist subject. The posthuman movement has been accompanied by a move towards new materialist theories and methodologies that seek to expose the ways in which im/material phenomena impact on and affect one another. One popular theoretical trope is that of the assemblage (e.g. Crook, 2011; Dewsbury, 2011; Saldanha, 2012; Price-Robertson and Duff, 2016). This concept positions the posthuman body and mind as one constituted by a mixing and merging of the organic and the manufactured, the given and the created, the physical and the virtual, the body and society, the human and the non-human (Goodley et al, 2021). One might utilise this idea to understand and, indeed, appreciate the global assemblage offered by power of digital spaces to reinvigorate People First and self-advocacy communities during lockdown (Cook et al, 2021) – as demonstrated by the organisations’ responses described above – and the positive impacts on health and well-being that are gained through the creation of new online imaginations, alliances and connectivity (Fritsch, 2015). And yet, alongside these opportunities, many disabled people have been marginalised by the move towards online medical consultations and forms of pandemic healthcare rationing that masquerade in the name of technological progress (Moloney et al, 2022). These experiences echo ‘the collective historical trauma in the disability community, in which people with disabilities are viewed as inherently ‘less than’ and disposable’ (Lund and Ayers, 2020: S210). As Lin and Yang (2020) argue, we need to counsel against uncritical views of the ‘digital promised land’ when human-technological couplings create new forms of cost-cutting governance, austerity and rationalisation. There has been a rise in scholarship exploring animal, machine, human and ecological co-dependencies (see for example in critical disability studies; Hemingway and Priestley, 2006; Pepper, 2007, 2018; Taylor, 2011, 2017; Reeve, 2012; Jenkins and Taylor, 2020; Lundblad, 2020; Teachman et al, 2020; Jammaers, 2021). What is clear from this work is that the many non-human elements of assemblages – like their human counterparts – are steeped in historical practices of exclusion and inclusion. As Haritaworn (2015: 211) cautiously puts it, ‘it is essential to interrogate the nonhuman alongside the dehumanization of ‘Man’s human Others’. These non-human others of animals, machines and the environment are all deeply marked by processes of racism/colonialism and ableism/disablism (see for brief and insightful piece, Hird, 2015). Practices and imaginaries of AI, avatars and robotics – the gleaming materialities of our brave new world of the medical posthumanities – are tarnished by built-in assumptions of able-bodied-and-mindness (McMillan, 2015; Kögel and Wolbring, 2020; Cave and Dihal, 2020). New materialist and posthuman speculations must beware of importing ahistorical and apolitical conceptualisations of the non-human: the more-than-human is not a benevolent offering by nature or design.

So where does this leave the more-than-human register? Braidotti (2019a: 53) writes that class, race, gender and sexual orientations, age and able-bodiedness continue to function as significant markers in framing and policing access to normal ‘humanity’. What is required, then, to counter this humanism is a Missing People’s Humanities, an expansive community of theory, arts and activism that spans multiple human and more-than-human alliances, one that

promotes the insurrection of women – as the others of ‘Man’ – and other ‘others’, like LBGT+, non-whites (postcolonial, black, Jewish, indigenous and native subjects) and non-humans (animals, insects, plants, tress, viruses, fungi, bacteria and technological automata) (Braidotti, 2019a: 39).

People First emerges in a world populated by a cacophony of activist voices and much work is to be done to explore their intersectional connections and frictional touching points. Some of these crossovers offer radical departures from the logics of humanism and, indeed, normative conceptions of the human. Here we will find theorisations that embrace the abject humanities as spaces of resistance and affirmation including bare life (Overboe, 2007); fugitivity and outlaws (Moten, 2008; Miller, 2020); mutualities with animal lives (Taylor, 2011; 2017); intracorporeal multiplicity (Fritsch, 2015); the inhuman (Hird, 2015) and the DisHuman (Goodley and Runswick-Cole, 2016). Clearly activism and social theory are moving in these posthuman directions; but a question remains: to what extent is disability a guiding subject of these intellectual transformations?

#### iii human troubles

This leads me to the final tension I want to address: *human troubles*. An uncritical move into the medical posthumanities risks ignoring the institutionalisation of human forms of ableism and ability privilege that not only mark but also *define* whose health and well-being is valued. Our contemporary times are tied to narrow conceptions of human progress in which certain non/humans suffer as the collateral damage of these wider socio-economic and cultural conditions. These contemporary times are underpinned by an ideology of neoliberal-ableism (Goodley, 2014; Goodley, Lawthom and Runswick-Cole, 2014; Goodley and Lawthom, 2019). Inspired by the work of disabled scholars such as Wolbring (2008, 2009, 2012), I am reminded of the powerful ways in which human conduct and ethics are guided and infiltrated by the ideology of ableism – a worldview that promotes the species typical individual human being – an ideology that inculcates as natural a model of the global citizen that is ready and able to work and productively contribute, an atomistic phenomenon bounded and cut off from others, capable, malleable and compliant (Goodley and Lawthom, 2019: 235). This species-typicality is at the centre of Wolbring’s work, drawing attention to the societal idealisation of a normative idea of what it means to be *homo sapiens* (the Latin words, by the way, for ‘wise man’) (Ibid). Neoliberalism is described by Ong (2007) as being the latest stage in capitalism’s global hegemonic domination. Like ableism, neoliberalism gets under our skin, inside our heads, and infects our relationships with non/human others, creating what Jakobsen (2009) describes as ‘a relational structure that provides for privatized resource-provision’. Together, then, *neoliberalism provides an ecosystem for the nourishment of ableism; which we can define as neoliberal-ableism*’ (Goodley, 2014: 34). Following Goodley and Lawthom (2019: 245), we must confront neoliberal–ableism’s psychological, social, economic, and cultural character. This is a mindset that privileges able-bodied and minded-ness, creates social spaces only fit for normative citizens, leads to institutional-bias towards autonomous bodies and minds, and encourages an economic dependence on the marketplace. Disabled bodies and minds struggle to flourish in this psycho-political context. Healthcare rationing is a direct manifestation of healthcare and political systems that routinely dehumanise disabled people. Medical posthumanities requires a reflexivity: to dig out and critique ableist and disablist assumptions and to counter the constitution of human troubles.

## Conclusions

This paper has made a case for critically engaging with the medical posthumanities through demonstrating the tensions that emerge as a consequence of considering an event and response from the perspective of a given field. On one level, I have concerns with this emerging scholarship if it fails to attend to dominant constructions of disability that continue to promulgate implicit understandings of the human and the more-than-human that are deeply ableist. The problem with able-bodied-and-mindness is that its presence is often assumed and rarely interrogated (Matias et al, 2014). Without interventions from disability scholars, there is a danger that ability-washing will afflict the work of the medical posthumanities. While sitting with this critique of others I also am aware of my own failings, especially the lack of explicit connection I have made with other transformative perspectives including BIPOC, LGBTQIIA+ and working-class communities (see for examples Razack, 2016; Byrd, 2020; Dolezal et al, 2021). Overall, in reflecting on this partial and brief critique of medical posthumanities, I feel propelled to identify a number of urgent projects:

- Unpacking the human kinds, assumptions and conceptualisations that underpin theorisation in the medical posthumanities.
- Identifying those humanities that are included or excluded by the medical posthumanities.
- Contesting human precarity, discrimination and oppression whilst recognising our more-than-human constitution.
- Combining a commitment to non-human animals, machines and environments and humans alongside a sensitivity to the different histories of oppression and exclusion that undergird these different elements of the posthuman condition.
- Addressing the tensions that are created when we affirm the non-human at a time when many human beings are engaged with redressing their negation as humans.
- Increasing the representation of disabled scholars in the medical humanities.

Along with Adams and Weinstein (2020: 235) I propose a positionality that is ‘in but not of the posthuman turn’, cognisant of our contemporary times that continue to render some human beings as valued and others as expendable. Specifically, I would argue that it is incumbent on all of us to ensure that disability moves from being an ‘absent presence’ to becoming a driving subject of inquiry.^5^

## Footnotes

2 I want to acknowledge here a sense of discomfort in writing lists such as these of ‘the disposed’ and ‘the dispossessed’. There is always the risk of excluding some, the question of ordering, and the dangers of conflating diverse human experiences. We might not be able to get away from lists but we recognise the need to be more self-conscious and aware of the discomfort that is created. I thank Katherine Runswick-Cole for alerting me to these dangers of list-making.

3 See for examples https://www.sheffield.ac.uk/ihuman/our-work/marginalised-humans/critical-disability-studies

4 I follow up and develop this argument in a forthcoming piece (Goodley, in press)

5 This aspiration is taken forward in a new international research project entitled Disability Matters that challenges the dominant disciplines of medical humanities, medical sociology, Science and Technology Studies to consider disability as the entry point into contemplations of theory, method and analysis – https://sites.google.com/sheffield.ac.uk/disability-matters/home
